# Editorial: Advances and challenges of non-invasive brain stimulation in age-related neurodegenerative diseases, volume II

**DOI:** 10.3389/fnagi.2023.1275530

**Published:** 2023-08-24

**Authors:** Yi Guo, Wei Wu, Junhong Zhou

**Affiliations:** ^1^Department of Neurology, Shenzhen People's Hospital, The Second Clinical Medical College, Jinan University, Shenzhen, Guangdong, China; ^2^The First Affiliated Hospital, Southern University of Science and Technology, Shenzhen, Guangdong, China; ^3^Shenzhen Bay Laboratory, Shenzhen, Guangdong, China; ^4^Alto Neuroscience Inc., Los Altos, CA, United States; ^5^Harvard Medical School, Hebrew Seniorlife Hinda and Arthur Marcus Institute for Aging Research, Roslindale, MA, United States

**Keywords:** brain stimulation, neurodegenerative disease, aging, cognition, clinical application

This editorial summarizes the contributions of the Frontiers Research Topic “*Advances and Challenges of Non-Invasive Brain Stimulation in Age-Related Neurodegenerative Diseases*”, highlighting the significance of aging as a risk factor for neurodegenerative diseases, the limited effectiveness of current treatments and the potential of non-invasive brain stimulation.

Aging is a major risk factor for neurodegenerative diseases. Neuronal losses during aging can lead to cognitive decline, particularly when combined with accumulation of toxic proteins. Treatment options for these conditions are limited. Noninvasive brain stimulation (NiBS) techniques, such as transcranial magnetic stimulation (TMS) and transcranial current stimulation (TCS), can modulate brain networks and enhance cognitive functions in both healthy individuals and neurodegenerative patients. The effects of NiBS can persist even after the stimulation ends. However, a comprehensive understanding of its biological mechanisms and clinical applications is still lacking, which poses challenges. This topic aims to explore NiBS as an innovative therapeutic tool, specifically discussing its advances and challenges in treating age-related neurodegeneration.

A study conducted by He et al. promoted that spinal cord stimulation (SCS) is a promising treatment for disorders of consciousness (DoC). This study analyzed 66 DoC patients who received SCS treatment to investigate the association between postoperative cerebrospinal fluid (CSF) protein levels and consciousness improvement. Patients with permanent electrodes had higher CSF protein levels than those with temporary percutaneous electrodes. Moreover, elevated CSF protein levels were linked to reduced sagittal diameter and poor outcomes at 3 months. The findings propose that reducing the influence of electrode pads on anatomical changes may improve treatment outcomes. CSF protein levels could serve as potential biomarkers of postoperative outcomes and deserve further exploration.

Another study (Ni et al.) evaluated the effects of various repetitive TMS (rTMS) stimulation procedures on upper limb function and brain functional network characteristics in stroke patients. The study involved 36 stroke patients who were assigned to either receive 1 Hz stimulation in the contralesional hemisphere and 10 Hz stimulation in the affected hemisphere, or solely 10 Hz stimulation in the affected hemisphere. The results demonstrated that rTMS treatment improved upper limb motor function, enhanced brain network connections, and reduced activation of isolated brain areas. Depending on the specific brain network states, optimal rTMS treatment plans could be suggested for precise rehabilitation.

Another study (Zheng et al.) explores the effects of continuous theta burst stimulation (cTBS) on enhancing language abilities in patients with aphasia. The researchers focused on targeting the right posterior superior temporal gyrus (pSTG), a region acknowledged for its critical role in semantic processing. This article presents findings from a randomized controlled trial involving 34 aphasic patients who underwent either cTBS or sham stimulation, followed by speech and language therapy. The study aimed to uncover whether cTBS applied to the right pSTG could promote language recovery and elucidate the underlying brain mechanisms. The results showed promising effects of cTBS, manifesting as improved language performance and modulation of brain activity and connectivity in specific regions. Overall, this research offers valuable insights into potential therapeutic interventions for language rehabilitation in individuals with aphasia.

One article (Bagattini et al.) investigates the effects of brain stimulation on two different age groups: middle-aged adults (40–60 years) and older adults (65 years and above). The researchers aimed to understand how tDCS impacts behavior and brain function differently in these two groups. Additionally, behavioral assessments and neurophysiological measurements were performed. This research provides valuable insights into age-specific brain stimulation effects and its potential applications for cognitive enhancement.

Another article (Xiong et al.) explores the potential therapeutic effects of two brain stimulation techniques, median nerve stimulation (MNS) and rTMS, on patients with prolonged disorders of consciousness (pDOC). In a rigorously conducted trial, 75 eligible patients with pDOC were randomly assigned to one of three groups: (1) rTMS + sham-MNS; (2) MNS + sham-rTMS; or (3) MNS + rTMS. The primary outcome was the change in the Coma Recovery Scale-Revised (CRS-R) score after treatment. Findings showed that the combined MNS + rTMS intervention yielded significantly greater improvements in Glasgow Coma Scale (GCS) scores and somatosensory evoked potentials, compared to the other two groups. This research offers valuable insights into the potential benefits of combining brain stimulation modalities to enhance consciousness recovery in patients with pDOC.

In a study conducted by Turrini et al., researchers investigated the effects of brain stimulation using ccPAS on two groups: young adults (20–35 years) and elderly adults (65–80 years). They aimed to measure the impact of stimulation on motor skills and cortex responsiveness. The study revealed significant age-related differences, indicating that ccPAS enhances action performance and corticomotor excitability more effectively in young adults compared to elderly adults. These findings offer valuable insights into the age-specific effects of brain stimulation and its potential applications for cognitive enhancement. By delving into this research, the researcher can gain deeper understanding of how brain stimulation techniques may differ in their outcomes based on age, contributing to advancements in neurostimulation and its implications for age-related cognitive functions.

Another study (Naparstek et al.) presents a comprehensive review of the potential uses of transcutaneous vagus nerve stimulation (tVNS), a non-invasive neuromodulation technique, in the context of cognitive aging. The authors have compiled a thorough review and provided valuable commentary on the existing literature, shedding light on the effectiveness of tVNS in enhancing various cognitive functions in older adults. Through this review, researchers can uncover the promising applications of tVNS for memory, attention, and executive functions in the aging population. This review not only synthesizes the current knowledge on tVNS but also suggests pathways for future research, offering new insights into innovative therapeutic approaches for addressing age-related cognitive decline.

## Author contributions

YG: Writing—original draft. WW: Writing—review and editing. JZ: Writing—review and editing.

